# Observation of Intact and Proteolytically Cleaved Amyloid-Beta (1–40)-Oleuropein Noncovalent Complex at Neutral pH by Mass Spectrometry

**DOI:** 10.3390/molecules26113261

**Published:** 2021-05-28

**Authors:** Ioana Cezara Caba, Raluca Ştefănescu, Bogdan Ionel Tamba

**Affiliations:** 1 Center for Advanced Research and Development in Experimental Medicine (CEMEX), “Grigore T. Popa” University of Medicine and Pharmacy, 16 Universitatii Street, 700115 Iasi, Romania; ioana-cezara.caba@umfiasi.ro (I.C.C.); bogdan.tamba@umfiasi.ro (B.I.T.); 2 Department of Toxicology, Faculty of Pharmacy, “Grigore T. Popa” University of Medicine and Pharmacy, 16 Universitatii Street, 700115 Iasi, Romania; 3 Department of Pharmacology, Clinical Pharmacology and Algesiology, “Grigore T. Popa” University of Medicine and Pharmacy, 16 Universitatii Street, 700115 Iasi, Romania

**Keywords:** Alzheimer’s disease, beta-amyloid peptide, oleuropein, mass spectrometry, noncovalent complex, electrospray ionization, triple quadrupole

## Abstract

Mass spectrometry analyses carried out on mass spectrometers equipped with soft ionization sources demonstrated their utility in the assessment of the formation of noncovalent complexes and the localization of the binding sites. Direct analyses by mass spectrometry of the noncovalent complex formed in acidic and mildly acidic environments by amyloid beta (1–40) peptide and oleuropein have been previously described, and, in several studies, the absorption, metabolism, excretion, and the implications in the prevention and therapy of Alzheimer’s disease of oleuropein have been investigated. Our paper presents modifications of the method previously employed for noncovalent complex observation, namely, the amyloid beta (1–40) pretreatment, followed by an increase in the pH and replacement of the chemical environment from ammonium acetate to ammonium bicarbonate. The formation of noncovalent complexes with one or two molecules of oleuropein was detected in all chemical solutions used, and the amyloid beta (17–28) binding site was identified via proteolytic experiments using trypsin prior to and after noncovalent complex formation. Our results highlight the importance of further studies on the effect of oleuropein against amyloid beta aggregation.

## 1. Introduction

The study of noncovalent complexes, and particularly the identification of molecules’ binding regions, is essential for biomedical studies, as it allows for the investigation of the interaction of different molecules in living organisms and the effects exerted by foreign molecules to which living organisms are exposed. Examples of such interactions are antigens–antibodies and receptors–ligands.

Mass spectrometry methods developed for noncovalent complex analysis are valuable tools for assessing, directly or indirectly, whether the tested substances interact. Direct analysis by mass spectrometry of complexes formed in a solution is possible by using a chemical medium similar to those found in the human physiology and ensuring the mass spectrometric compatibility of the chemical solutions containing the molecules whose interactions are studied. Both electrospray ionization (ESI) and matrix assisted laser desorption and ionization (MALDI) were proven to be effective ionization methods for the analysis of noncovalent complexes and of compounds belonging to different classes of molecules; however, studies aimed at analyzing noncovalent complexes at neutral pH using MALDI are less frequent [1,2]. The indirect methods involve the mass spectrometric identification of a compound exposed to an affinity medium containing the immobilized cognate biomolecule and eluted using a dissociating solution that is compatible with electrospray ionization (ESI) or matrix assisted laser desorption and ionization (MALDI). The latter approach allows the selection of a chemical medium which contains inorganic ions and biomolecules resembling the body chemical environment in which the interaction takes place.

Alzheimer’s disease (AD) is an uncurable neurodegenerative illness. According to the amyloid cascade hypothesis, abnormal extracellular accumulation of aggregated amyloid beta peptides in the brain leads to neurotoxicity and cognitive decline.

Amyloid beta (Aβ) peptides are naturally occurring amino acid chains produced from an amyloid precursor protein (APP) by a cleavage pathway involving two proteases: beta- and gamma-secretase. The resulting peptides, denoted as Aβ(1–40) and Aβ(1–42), contain mainly 40 or 42 amino acids and represent the amyloid precursor protein fragments APP(672−711) and APP(672−713), respectively. Amino-terminal truncated peptides were also reported. The role of amyloid beta in the human body is not yet completely understood. It was proven that the ratio of Aβ(1–40) to Aβ(1–42) is lower in patients with inherited Alzheimer’s disease forms in comparison to healthy individuals [3].

Passive and active immunotherapeutic approaches targeting the clearance of amyloid beta peptides from body fluids have been investigated in recent years. Mass spectrometry, affinity chromatography, and proteolytic cleavage have been proven to be effective methods for epitope elucidation. Epitope excision and extraction of the amino acid sequence from amyloid beta peptides recognized by the amyloid beta specific antibodies were employed in several studies. Similar studies were carried out for the identification of the amyloid precursor protein epitope of an antibody directed towards the carboxyl-terminal end of the amyloid precursor protein. Furthermore, mass spectrometry methods for the identification of paratopes and complete antibody sequencing of human autoantibodies recognizing beta amyloid peptide were developed [4,5].

Considering that one of the current approaches for preventing Alzheimer’s disease aims to identify the aggregation inhibitor of an amyloid beta peptide, the current paper investigates the interaction, at neutral pH, between the Aβ(1–40) peptide and the glycosylated secoiridoid oleuropein, a secondary metabolite present in high amounts in olive tree leaves (Olea europaea) [6]. The observation method of the noncovalent complex at pH 3.0 was previously developed by Bazoti et al. [7]. The authors found two molecules of oleuropein binding to one molecule of Aβ(1–40) and investigated the binding sequence interacting with oleuropein by proteolytic cleavage prior to, or after, the addition of oleuropein, followed by direct analysis of the resulting peptide mixture. The results showed that the sequence of Aβ(17–28) represents one of the sequences of amyloid beta peptide involved in the binding to oleuropein [8]. Compared with previous published studies, our paper introduces the following methodology changes: a) a modified method for the pretreatment and solubilization of amyloid beta peptide (1–40) and b) the analysis of the noncovalent complex and identification of the binding sequence at different pH values, including neutral pH (7.35).

## 2. Results and Discussions

### 2.1. Preparation of Beta-Amyloid (1–40) and Oleuropein Stock Solutions

Beta-amyloid (1–40) was solubilized in 1,1,1,3,3,3-hexafluoroisopropanol at 1 mg/mL concentration, according to Yoshiike et al. [9], with incubation for 2 h, at room temperature, to preserve the monomeric form of the peptide. Considering the effect of fluorinated alcohol in inducing alpha-helical conformation at peptides and proteins, the peptide Aβ(1–40) was allowed to dry prior to the addition of the solvent and oleuropein for the investigation of noncovalent complex formation.

Based on the study published by Yateem et al. [10], oleuropein was solubilized in 20% acetonitrile and 80% double-distilled water. 

### 2.2. Complex Formation at pH 3

**Figure 1:**
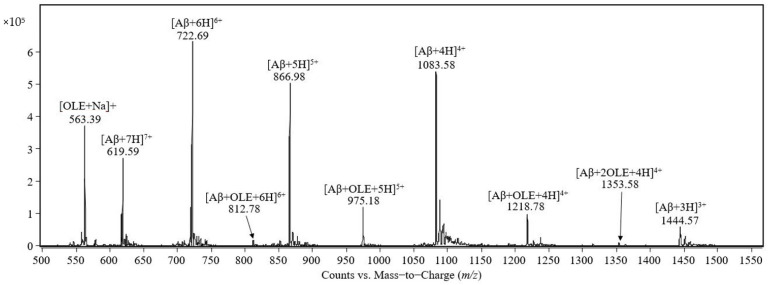
Mass spectrum of the 1:1 complex formed between Aβ(1–40) and oleuropein both at 50 pmol/μL in a final solution of 0.5 mM ammonium acetate and 0.25% acetic acid, pH 3, recorded 30 min after incubation on the electrospray-triple quadrupole mass spectrometer.

The mass spectrum of the equimolar mixture of Aβ(1–40) peptide and oleuropein prepared in 0.5 mM ammonium acetate containing 0.25% acetic acid, pH 3, is shown in Figure 1. The signals at *m*/*z* 1444.57, 1083.58, 866.98, 722.69, and 619.59 were assigned to the molecular ions of the Aβ(1–40) molecules protonated with 3, 4, 5, 6, and 7 protons, respectively. The signal at *m*/*z* 563.39 was assigned to the sodium adduct of oleuropein [OLE + Na]^+^. The peaks observed at *m*/*z* 1218.78, 975.18, and 812.78 were assigned to the complexes between one molecule of Aβ(1–40) and one molecule of oleuropein protonated with four, five and six protons, respectively. The peak observed at *m*/*z* 1353.58 was assigned to the quadruple-protonated molecular ion of the complex formed between Aβ(1–40) and two molecules of oleuropein. The signal at *m*/*z* 1624.47 was assigned to the complex between Aβ(1–40) and one molecule of oleuropein protonated with three protons and was observed by examining the *m*/*z* range 1500–2000 (result not shown in the Figure 1) and, at *m*/*z* 1804.57, the triple-protonated molecular ion of Aβ(1–40) forming a complex with two molecules of oleuropein was observed.

### 2.3. Complex Formation in Ammonium Acetate at Different pH Values

The analysis by electrospray-triple quadrupole of four solutions containing 50 pmol/μL Aβ(1–40) solubilized in ammonium acetate 0.5 mM at pH values of 4.4, 5.3, 6.2, and 7.4 indicates that all four mass spectra signals are present at *m*/*z* 1083.48, 866.98, 722.69, and 619.59 corresponding to Aβ(1–40) molecules possessing 4, 5, 6 and 7 positive charges, respectively, denoted as [Aβ + 4H]^4+^, [Aβ + 5H]^5+^, [Aβ + 6H]^6+^, [Aβ + 7H]^7+^. In the next step, mass spectra were acquired for the solutions containing 50 pmol/μL Aβ(1–40) and 100 pmol/μL oleuropein solubilized in 0.47 mM ammonium acetate at four pH values (4.4, 5.3, 6.2, 7.4). In all four mass spectra, at the *m*/*z* values 1083.48, 866.98, 722.69, and 619.59, there are signals corresponding to molecules of Aβ(1–40) carrying 4, 5, 6, and 7 protons. The signal at *m*/*z* 563.39 corresponds to the adduct with sodium of one molecule of oleuropein (denoted as [OLE + Na]^1+^) and the signals observed at *m*/*z* 975.18 and 1218.68 correspond to the noncovalent complexes formed between one molecule of Aβ(1–40) and one molecule of oleuropein carrying 5 and 4 protons, respectively ([Aβ + OLE + 5H]^5+^, [Aβ + OLE + 4H]^4+^). A similar result was obtained for the samples incubated for 2 h at 25 ℃.

**Figure 2:**
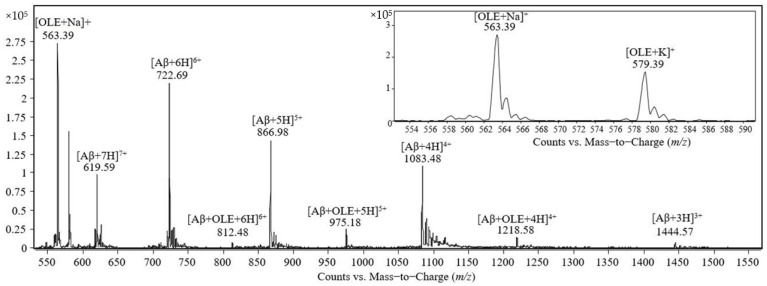
Mass spectrum obtained by ESI-triple quadrupole analysis of the equimolar solution containing Aβ(1–40) and oleuropein at 50 pmol/μL in 0.47 mM ammonium acetate, pH 7.4, after incubation for 30 min at room temperature.

The mass spectrum obtained after the analysis of the equimolar solution containing Aβ(1–40) and oleuropein at the concentration of 50 pmol/μL in 0.47 mM ammonium acetate, pH 7.4, is shown in Figure 2. The peaks observed in the mass spectrum were identified as follows: (i) the peaks at *m*/*z* 563.39 and 579.39 correspond to the adducts with sodium and potassium, respectively, denoted as [OLE + Na]^1+^ and [OLE + K]^1+^; (ii) the peaks at *m*/*z* 1444.57, 1083.48, 866.98, 722.69 and 619.59 correspond to Aβ(1–40) molecules carrying 3, 4, 5, 6, and 7 positive charges, respectively; (iii) the peaks at *m*/*z* 1218.58, 975.18, and 812.48 correspond to the noncovalent complex formed by one molecule of Aβ(1–40) and one molecule of oleuropein protonated with 4, 5, and 6 protons, respectively.

### 2.4. Complex Formation in Ammonium Bicarbonate at Neutral pH

**Figure 3:**
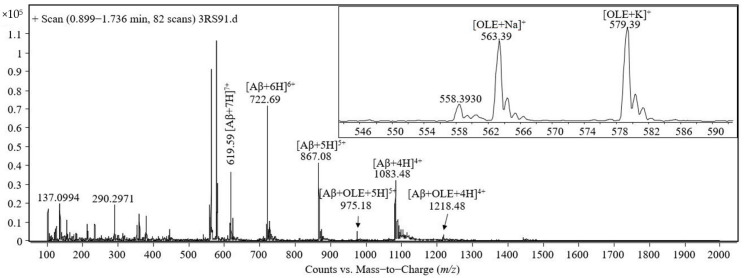
Mass spectrum obtained by ESI-triple quadrupole analysis of the equimolar solution containing 50 pmol/μL Aβ(1–40) and oleuropein in 1 mM ammonium bicarbonate, pH 7.35, incubated for 30 min at 37 ℃.

The mass spectrum acquired during the analysis at ESI-triple quadrupole of the solution containing equimolar amounts of Aβ(1–40) and oleuropein at the concentration of 50 pmol/μL, incubated for 30 min at 37 ℃, is presented in the Figure 3. A second analysis was performed for the same sample, which was agitated at 37 ℃ for 2 h and a mass spectrum was recorded. The mass spectrum presented in the Figure 3 contains the same signals as described above for Figure 2.

### 2.5. Enzymatic Cleavage of Aβ(1–40) Prior to and after Complex Formation

**Figure 4:**
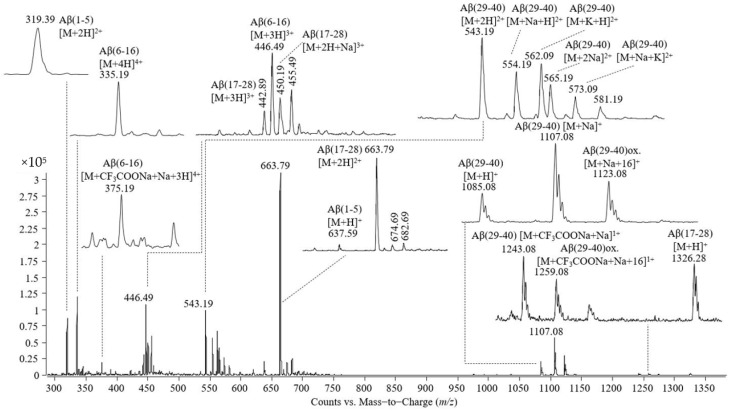
Mass spectrum obtained by ESI-triple quadrupole analysis of Aβ(1–40), proteolitically cleaved by trypsin.

**Figure 5:**
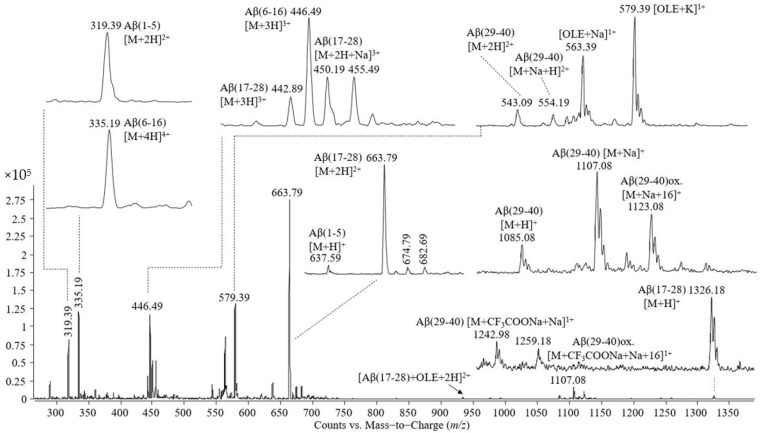
Mass spectrum obtained by ESI-triple quadrupole analysis of the proteolytic mixture resulting from Aβ(1–40) cleavage by trypsin, incubated with oleuropein for 30 min at 37 °C.

In the next step, we carried out an experiment for the identification of the Aβ(1–40) amino acid sequence with the minimal amino acid residue length essential for oleuropein binding. The Aβ(1–40) peptide was first proteolytically cleaved by trypsin and the mass spectrum, as per Figure 4, was acquired; then, the peptide mixture was allowed to interact with oleuropein, followed by the analysis at electrospray-triple quadrupole. The mass spectrum recorded is shown in Figure 5.

The peaks observed in the mass spectrum shown in Figure 4 are as follows: (i) single- and double-protonated molecular ions of the peptide Aβ(1–5) at *m*/*z* values of 637.59 and 319.39; (ii) triple- and quadruple-protonated molecular ions of the peptide Aβ(6–16) and the adduct with trifluoroacetate and sodium carrying four positive charges at *m*/*z* 446.49, 335.19, and 375.19, respectively; (iii) single-, double-, and triple-protonated molecular ions of the peptide Aβ(17–28) at *m*/*z* 1326.28, 663.79, and 442.89, respectively, and the adduct with one atom of sodium of the triple charged molecular ion of the peptide Aβ(17–28); (iv) a single-protonated molecular ion of the peptide Aβ(29–40) at *m*/*z* 1085.08, the adduct with sodium at *m*/*z* 1107.08, the adduct with sodium of the Aβ(29–40) oxidized at *m*/*z* 1123.08; (v) a double-protonated molecular ion of the peptide Aβ(29–40) at *m*/*z* 543.19, the adducts of this peptide with one atom of sodium or potassium at *m*/*z* 554.19 and 562.09, the double-cationized molecular ion of Aβ(29–40), namely, cationization with two atoms of sodium at *m*/*z* 565.19, cationization with one atom of sodium and one atom of potassium at *m*/*z* 573.09; (vi) singly charged adducts with trifluoroacetate and sodium formed by Aβ(29-40) in non-modified and oxidized states.

In addition to these molecular ions, the mass spectrum presented in Figure 5 contains signals at *m*/*z* 563.39 and 579.39, corresponding to the single charged adducts with sodium and potassium of oleuropein and the peak at *m*/*z* 933.98 corresponding to the noncovalent complex formed between the peptide Aβ(17–28) and oleuropein identified as a double-protonated molecular ion.

**Figure 6:**
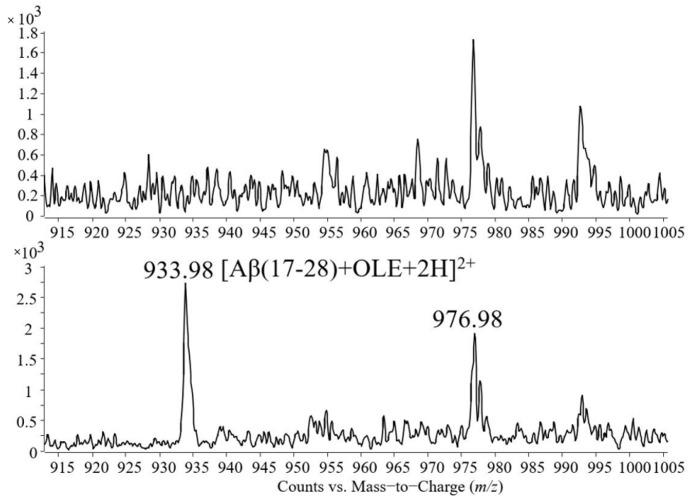
Detailed view of the *m*/*z* range 915–1005 and comparison between the sample containing Aβ(1–40) tryptic mixture presented in Figure 4 and the sample containing Aβ(17–28)-oleuropein noncovalent complex presented in Figure 5.

Figure 6 shows a detailed representation of the *m*/*z* range 915–1005. The spectrum placed in the upper part of the figure contains the signals present in the sample containing Aβ(1–40) cleaved by trypsin, while the spectrum placed in the lower part contains the signals of the tryptic mixture incubated with oleuropein.

In the next step, the Aβ(1–40) peptide and oleuropein were allowed to form noncovalent complexes and, after confirming the formation of the complex via mass spectrometry, trypsin was added to the sample and allowed the chain of the peptide Aβ(1–40) to be cleaved. The mass spectrum showed the same fragment Aβ(17–28) binding to one molecule of oleuropein.

The identification of the binding site was further investigated using the proteolytic enzyme GluC. Proteolysis of Aβ(1–40) by GluC prior to and after the formation of the noncovalent complex led to the observation of numerous molecular ions corresponding to proteolytic peptide fragments. However, the fractions that should contain the noncovalent complex between oleuropein and one of the proteolytic peptide fragments resulting from the cleavage of Aβ(1–40) peptide by GluC indicated the absence of a noncovalent complex.

Table 1 contains the assignments of the peaks observed in the mass spectra presented.

## 3. Materials and Methods

### 3.1. Materials

Aβ(1–40) peptide with an amidated carboxyl-terminal end, prepared as trifluoroacetate salt, was purchased from Bachem AG, Bubendorf, Switzerland (H−7664.1000). Oleuropein, 1,1,1,3,3,3-hexafluoro-2-propanol (HFIP) and ammonium bicarbonate were purchased from Sigma Aldrich, Saint Louis, MO, USA. Ammonium acetate was purchased from Honeywell Speciality Chemicals Seelze GmbH, Seelze, Germany. Acetonitrile and water (HPLC purity) were purchased from LabScan analytical sciences, Gliwice, Poland. Formic acid was purchased from Merck, Darmstadt, Germany, and acetic acid was purchased from Lachner, Czech Republik. Double-distilled water was produced by an Autostill^TM^ apparatus distributed by Jencons Scientific Ltd, Leighton Buzzard, England.

### 3.2. Preparation of Stock Solutions of Aβ(1–40) and Oleuropein

The vial containing lyophilized powder Aβ(1–40) peptide was stored upon receiving shipment until use in the freezer at −24 °C. For the preparation of Aβ(1–40) stock solution, the tube was first allowed to reach room temperature. To ensure the location at the bottom of the vial of the entire amount of peptide (1 mg), the vial was centrifuged for 30 s at 3000 rpm. An amount of 1 mg of Aβ(1–40) peptide was solubilized in 200 μL of 100% HFIP and the vial was agitated using a vortex 3 times for 30 s, centrifuged for 30 s at 3000 rpm, and the solution was transferred into a Protein LoBind tube. The remaining Aβ(1–40) was washed 4 times with 200 μL 100% HFIP, repeating the agitating and centrifugation steps, and all five fractions were mixed. The tube was spun for 30 min at 700 rpm and 23 °C. The resulting solution, having the final concentration 1 mg/mL of Aβ(1–40), was stored at −24 °C. Prior to use, the tube containing Aβ(1–40) stock solution was allowed to reach room temperature.

Upon receiving the shipment, the vial containing 10 mg oleuropein was placed at 2 °C and stored until the preparation of the stock solution. Prior to use, the oleuropein was solubilized to a final concentration of 1 mg/mL in a solution containing 20% acetonitrile and 80% double-distilled water. The resulting solution was aliquoted in tubes containing 1 mL of oleuropein solution and placed at −24 °C for long-term storage, except for one aliquot placed at 2 °C for immediate and short-term use.

### 3.3. Complex Formation at pH 3

An amount of 64.9 μL of Aβ(1–40) stock solution was pipetted in a 1.5 mL Protein LoBind tube. The tube was agitated with the lid open at 600 rpm and at 24 °C for 2 h in an Eppendorf thermomixer F1.5. 150 μL of bi-distilled water were added to the dried film of Aβ(1–40) peptide located at the bottom of the tube. The sample was agitated in an Eppendorf thermomixer at 600 rpm and 24 °C for 5 min and mixed with 150 μL of 100 pmol/μL oleuropein solution prepared by diluting oleuropein stock solution with 1 mM ammonium acetate containing 0.5% acetic acid.

### 3.4. Complex Formation in Ammonium Acetate at Different pH Values

An amount of 10 μL of Aβ(1–40) stock solution was pipetted in each of four 1.5 mL Protein LoBind tubes and the HFIP was allowed to evaporate from the sample by agitating the tubes with the lids open at 600 rpm and 24 °C for two hours. Four solutions of 0.5 mM ammonium acetate with pH values of 4.4, 5.3, 6.2, and 7.4 were prepared by diluting a 10 mM stock solution of ammonium acetate and adjusting the pH with 10% acetic acid. Further, four solutions of 0.473 mM ammonium acetate containing 100 pmol/μL oleuropein were obtained, from which 46 μL were added to the four vials containing 10 μg of dried amyloid beta (1–40) peptide. The samples were stirred for 30 s using a vortex and 4.5 min using a thermomixer at 1500 rpm and were incubated at room temperature for two hours while spinning at 600 rpm. At 30 min and two hours, the samples were analyzed using the LC–MS system. The experiments were repeated, maintaining constant the concentration of Aβ(1–40) peptide and modifying oleuropein concentration for obtaining a molar ratio of 1:1, 1:0.5, and 1:0.25.

### 3.5. Complex Formation in Ammonium Bicarbonate at Neutral pH

An amount of 10 μL of oleuropein stock solution was diluted with 360 μL of 0.5 mM ammonium bicarbonate and, respectively, 360 μL of 1 mM ammonium bicarbonate. In total, 46.2 μL of each of these solutions was added separately to two Protein LoBind tubes containing 10 μg of Aβ(1–40). The samples were agitated for 30 s using a vortex and 4.5 min using a thermomixer at 1500 rpm and were incubated at room temperature for two hours while agitating at 600 rpm. After 30 min and after two hours of incubation, the samples were analyzed using the LC–MS system.

### 3.6. Identification of the Binding Sequence of Aβ(1–40) by Proteolytic Cleavage of Aβ(1–40) Prior to Complex Formation

An amount of 20 μL of beta-amyloid (1–40) stock solution was introduced into a 1.5 mL Protein LoBind vial and the sample was agitated for 75 min with the lid open at room temperature and 600 rpm. In total, 92.4 μL of 1 mM ammonium bicarbonate, pH 7.35, was added and the vial was agitated for 30 min at 37 °C. Then, 1 μL of a 1 μg/μL stock solution of trypsin solubilized in 50 mM acetic acid was added. The sample was agitated for 2 h at 37 °C and 600 rpm and 20 μL was removed.A volume of 10 μL was analyzed at the electrospray-triple quadrupole. The remaining 72.4 μL of the sample was mixed with 2.5 μL of oleuropein stock solution and the vial was incubated for 18 h at 37 ℃. At 30 min, two hours, and 18 h, 20 μL of the sample was removed and 10 μL was analyzed using the mass spectrometer.

### 3.7. Identification of the Binding Sequence of Aβ(1–40) by Proteolytic Cleavage of Aβ(1–40) after Complex Formation

Aβ(1–40) was incubated for 30 min with oleuropein in 1 mM ammonium bicarbonate, pH 7.35, with both substances at a concentration of 50 pmol/μL. The formation of the Aβ(1–40)–oleuropein complex was verified by analyzing 10 μL of the mixture. Due to the observation of noncovalent complexes, the sample was further mixed with trypsin for two hours at 37 °C and 10 μL was injected into the mass spectrometer.

### 3.8. Sample Analysis by LC-ESI-Triple Quadrupole Mass Spectrometer

The sample was injected directly into the 6410 electrospray-triple quadrupole mass spectrometer (Agilent Scientific) using an Agilent 1200 high performance liquid chromatograph by removing the chromatography column and connecting the tubing in the chromatography column compartment using a stainless-steel union. The solvents employed at the liquid chromatograph were solvent A, containing 5% acetonitrile and 0.1% formic acid in water, and solvent B, containing 80% acetonitrile and 0.1% formic acid in water. Solvents A and B were mixed in the mixing chamber in the ratio of 6.7% solvent B and 93.3% solvent A. The resulting solution contained 10% acetonitrile and 0.1% formic acid in water. The volume of sample injected was 10 μL and the flow rate was 0.05 mL/min.

The electrospray-triple quadrupole mass spectrometer was operated in positive mode. MS2 scans were carried out in the mass range of *m*/*z* 100–2000. Source conditions were: nebulizer pressure—35 psi, drying gas temperature—325 °C, capillary voltage—3850 V.

### 3.9. Data Analysis

The *m*/*z* values of the signals observed in the mass spectra, corresponding to intact molecules of oleuropein and beta-amyloid (1–40) peptide and to the cleavage peptide fragments resulting from the digestion of beta-amyloid (1–40) peptide by trypsin and endoprotease GluC, were compared to the *m*/*z* values calculated using an Excel spreadsheet prepared by the authors using the monoisotopic and average atomic masses of the chemical elements occurring in the chemical compounds present in the samples analyzed.

## 4. Conclusions

The modification of the sample pretreatment introduced in the method for the observation of beta-amyloid–oleuropein noncovalent complex at pH 3, published previously by Bazoti et al., allowed the formation of a noncovalent complex. Furthermore, by increasing the pH to 7.35, the noncovalent complex was identified in ammonium acetate and ammonium bicarbonate. Proteolytic cleavage by trypsin carried out in ammonium bicarbonate led to the identification of beta-amyloid (17–28) as the minimal amino acid sequence involved in noncovalent complex formation. The fragments beta-amyloid (12–22) and (23–40), resulting after a complete proteolytic cleavage by GluC of Aβ(1–40) in ammonium bicarbonate, do not separately preserve the affinity for oleuropein.

Due to the higher tendency of the peptide Aβ(1–42) to aggregate in comparison with Aβ(1–40), further studies should include the verification of Aβ(1–42)–oleuropein noncovalent complex formation. The stability of each noncovalent complex in human serum should be investigated and might be separated by immunoaffinity capture followed by observation by mass spectrometry.

## Figures and Tables

**Table 1:** Assignment of the signals observed in the mass spectra.

Molecular Ion		Analyzed Mass	Calculated *m*/*z*	Experimental *m*/*z*	Δm (Da)
Oleuropein monomer	[OLE + H]^+^	monoisotopic	541.19	-	-
	[OLE + Na]^+^	monoisotopic	563.50	563.39	0.11
	[OLE + K]^+^	monoisotopic	579.61	579.39	0.22
Aβ(1–40) Monomer	[Aβ + 7H]^7+^	average	619.41	619.59	−0.18
	[Aβ + 6H]^6+^	average	722.48	722.69	−0.21
	[Aβ + 5H]^5+^	average	866.77	866.98	−0.21
	[Aβ + 4H]^4+^	average	1083.21	1083.58	−0.37
	[Aβ + 3H]^3+^	average	1443.95	1444.57	−0.62
Noncovalent Complex	[Aβ + OLE + 6H]^6+^	average	812.56	812.78	−0.22
	[Aβ + OLE + 5H]^5+^	average	974.87	975.18	−0.31
	[Aβ + OLE + 4H]^4+^	average	1218.34	1218.78	−0.44
	[Aβ + 2OLE + 4H]^4+^	average	1353.47	1353.58	−0.11
	[Aβ + OLE + 3H]^3+^	average	1624.12	1624.47	−0.35
	[Aβ + 2OLE + 3H]^3+^	average	1804.30	1804.57	−0.27
Tryptic Fragment Aβ(1–5)	[M + 2H]^2+^	average	319.33	319.39	−0.06
	[M + H]^+^	monoisotopic	637.29	637.59	−0.30
Tryptic Fragment Aβ(6–16)	[M + 4H]^4+^	average	335.10	335.19	−0.09
	[M + CF3COONa + Na + 3H]^4+^	average	374.59	375.19	−0.60
	[M + 3H]^3+^	average	446.46	446.49	−0.03
Tryptic Fragment Aβ(17–28)	[M + 3H]^3+^	average	442.83	442.89	−0.06
	[M + 2H + Na]^3+^	average	450.15	450.19	−0.04
	[M + 2H]^2+^	average	663.74	663.79	−0.05
	[M + H]^+^	monoisotopic	1325.67	1326.28	−0.61
Tryptic Fragment Aβ(29–40)	[M + 2H]^2+^	average	543.69	543.19	0.50
	[M + Na + H]^2+^	average	554.68	554.19	0.49
	[M + K + H]^2+^	average	562.73	562.09	0.64
	[M + 2Na]^2+^	average	565.67	565.19	0.48
	[M + Na + K]^2+^	average	573.72	573.09	0.63
	[M + H]^+^	monoisotopic	1085.63	1085.08	0.55
	[M + Na]^+^	monoisotopic	1107.62	1107.08	0.54
	[M + Na + 16]^+^	monoisotopic	1123.61	1123.08	0.53
	[M + CF3COONa + Na]^+^	monoisotopic	1243.59	1243.08	0.51
	[M + CF3COONa + Na + 16]^+^	monoisotopic	1259.59	1259.08	0.51
Aβ(17–28)-Oleuropein Noncovalent Complex	[Aβ(17–28) + OLE + 2H]^2+^	average	934.00	933.98	0.02

## Sample Availability

Samples of the compounds are available from the authors.
